# Investigations on Zinc Isotope Fractionation in Breast Cancer Tissue Using *in vitro* Cell Culture Uptake-Efflux Experiments

**DOI:** 10.3389/fmed.2021.746532

**Published:** 2022-01-20

**Authors:** Kathrin Schilling, Adrian L. Harris, Alex N. Halliday, Christopher J. Schofield, Helen Sheldon, Syed Haider, Fiona Larner

**Affiliations:** ^1^Lamont Doherty Earth Observatory, Columbia University, Palisades, NY, United States; ^2^Department of Medical Oncology, Molecular Oncology Laboratories, Weatherall Institute of Molecular Medicine, University of Oxford, Oxford, United Kingdom; ^3^Department of Earth Sciences, University of Oxford, Oxford, United Kingdom; ^4^Department of Chemistry, University of Oxford, Oxford, United Kingdom; ^5^The Breast Cancer Now Toby Robins Breast Cancer Research Centre, The Institute of Cancer Research, London, United Kingdom

**Keywords:** Zn isotopes, MDA-MB-231, uptake, efflux, breast cancer, ZIP

## Abstract

Zinc (Zn) accumulates in breast cancer tumors compared to adjacent healthy tissue. Clinical samples of breast cancer tissue show light Zn isotopic compositions (δ^66^Zn) relative to healthy tissue. The underlying mechanisms causing such effects are unknown. To investigate if the isotopic discrimination observed for *in vivo* breast cancer tissue samples can be reproduced *in vitro*, we report isotopic data for Zn uptake-efflux experiments using a human breast cancer cell line. MDA-MB-231 cell line was used as a model for triple receptor negative breast cancer. We determined Zn isotope fractionation for Zn cell uptake (Δ^66^Zn_uptake_) and cell efflux (Δ^66^Zn_efflux_) using a drip-flow reactor to enable comparison with the *in vivo* environment. The MDA-MB-231 cell line analyses show Zn isotopic fractionations in an opposite direction to those observed for *in vivo* breast cancer tissue. Uptake of isotopically heavy Zn (Δ^66^Zn_uptake_ = +0.23 ± 0.05‰) is consistent with transport via Zn transporters (ZIPs), which have histidine-rich binding sites. Zinc excreted during efflux is isotopically lighter than Zn taken up by the cells (Δ^66^Zn_efflux_ = −0.35 ± 0.06‰). The difference in Zn isotope fractionation observed between *in vitro* MDA-MB-231 cell line experiments and *in vivo* breast tissues might be due to differences in Zn transporter levels or intercellular Zn storage (endoplasmic reticulum and/or Zn specific vesicles); stromal cells, such as fibroblasts and immune cells. Although, additional experiments using other human breast cancer cell lines (e.g., MCF-7, BT-20) with varying Zn protein characteristics are required, the results highlight differences between *in vitro* and *in vivo* Zn isotope fractionation.

## Introduction

Over the last decade analysis of natural metal isotopes has emerged as an interdisciplinary field of substantial biomedical potential, including diagnosis and defining disease mechanisms (1–14). Compared to metabolic studies where radioactive and single isotope tracers are used to monitor concentration changes, high precision measurements of natural isotopic fractionations are especially useful because they can inform on mechanisms, e.g., why changes in concentration occur due to altered uptake, secretion or excretion. Two recently published breast cancer studies found that Zn dyshomeostasis linked to carcinogenesis is reflected in enrichment of light Zn isotopes in malignant breast tissue compared to adjacent histologically healthy tissue (3, 5).

Zinc homeostasis in humans is maintained by multiple proteins which tightly regulate intracellular Zn concentrations. Among these proteins are influx-controlling Zn importers (SLC39A; ZIP1-ZIP14), efflux-controlling Zn transporters (SLC30A; ZnT1-ZnT10), and Zn-sequestering proteins (e.g., metallothionein) [e.g., (15–19)]. Histidine-, glutamate-, aspartate- and cysteine-residues serve as binding ligands for Zn in these proteins. In general, histidine-rich loops form the primary Zn-binding sites of ZIPs and ZnTs (20), while Zn-binding on metallothionein occurs on cysteine-rich ligands (21, 22). Cancer-induced Zn dyshomeostasis has been related to up- or down-regulation of Zn proteins (23) and changes in their coordination and ligand chemistry (24). For breast cancer, upregulation of ZIP6, ZIP7, ZIP10, ZnT2 and overexpression of metallothionein have been proposed to implicate higher Zn levels in malignant relative to healthy tissue (25–29).

Isotopic fractionation of Zn isotopes in cells is linked to coordination chemistry. Lower atomic weight is associated with lower bond energy, so heavier isotopes are enriched in the strongest ligand bonds, assuming equilibrium isotope fractionation (30–33). Thus, cysteine (S-ligands) preferentially binds isotopically light Zn, while normally tighter binding histidine (N-ligands) and aspartate (O-ligands) preferentially complex isotopically heavy Zn (30–33). If the normal Zn-ligand binding environments are disturbed by Zn dyshomeostasis, isotopic compositions in various body reservoirs (e.g., blood, urine, tissue) can be perturbed. Thus, Zn isotope analysis of clinical samples could be a method to probe the role of Zn uptake and secretion in cancer development and progression (1–3, 5, 11, 34).

*In vitro* cell lines are powerful simplified models to study processes at the molecular and cellular levels including the study of cancer-induced isotopic fractionation. Various human cell lines have been used to better understand uptake and transport mechanisms causing isotope fractionation of copper (35, 36), iron (37), uranium (38, 39) and Zn (38). Differentiated neuron-like cells (SH-SY5Y) preferentially incorporate and accumulate light isotopes of copper, uranium, and Zn (36, 38). Similar observations have been made for differentiated intestinal (Caco-2) cells showing the preferential uptake of isotopically light iron (37). Under oxidative stress, however, cancerous liver cells incorporate copper that is isotopically heavy (35, 40). These observations contradict the expected correlation between *in vitro* and *in vivo* studies on metal ion isotope fractionation.

**Figure 1:**
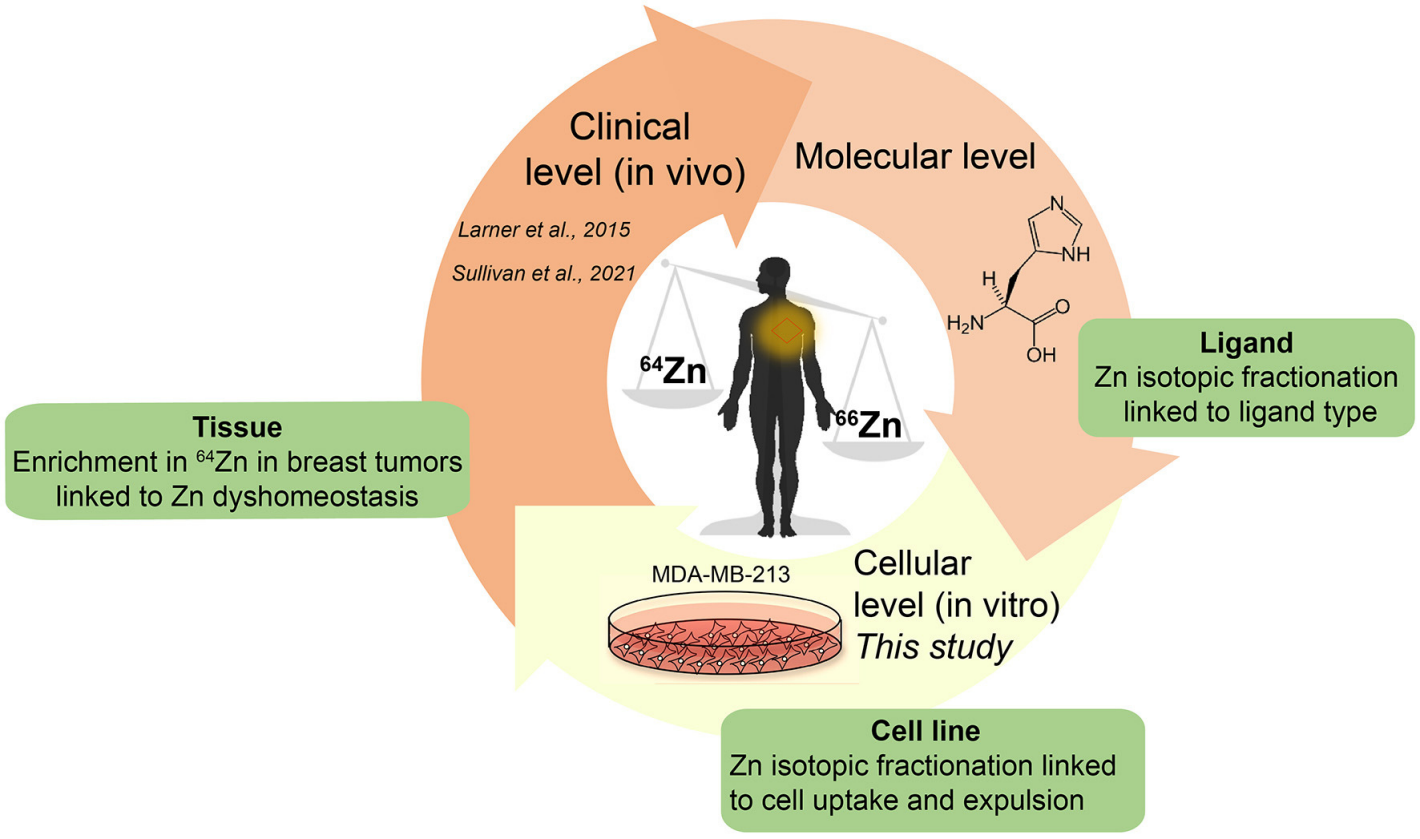
Schematic of overall approach for biomedical studies to investigate metal stable isotope fractionation.

There is little information about the processes at the molecular and cellular level causing systematic enrichment of light Zn isotopes in malignant breast tissue (3, 5). Our work aimed to improve the mechanistic understanding of the isotopic behavior of Zn in breast cancer, with the long-term objective of biomarker development. We conducted experiments using *in vitro* breast cancer cell lines (MDA-MB-231) spiked with isotopically-natural Zn and determined the isotopic fractionation induced due to active uptake and efflux. The results highlight differences between *in vitro* and *in vivo* Zn isotope processing and will inform future studies on using Zn isotope fractionation as a biomarker (Figure 1).

## Methods

### Cell Proliferation

The cell proliferation experiments were conducted to investigate if isotopic mass controls cell growth. Isotopically enriched metal powders of ^64^Zn (>99.00 %) and ^68^Zn (>99.00 %) (Isoflex, San Francisco, CA, US) were dissolved separately in 6 M HCl. Chloride solutions were diluted with 18 MΩ cm purified H_2_O (MilliQ) to make 10 mM ZnCl2 solutions which were then added to the exposure medium. The exposure medium consisting of Dulbecco's modified Eagle's medium (DMEM) + 10% fetal calf serum (FBS, Gibco Life, heat inactivated) + 1% penicillin/streptomycin (Sigma-Aldrich, UK) + 10 mM ZnCl_2_ solution was combined, adjusted to pH 7.4 if necessary and then equilibrated overnight. This exposure medium was added over two passages of MDA-MB-231 cells to enable full isotopic equilibration. At a cell density of 1 × 10^5^, cells were seeded in six well-plates (*n* = 4) for negative control (0 μM Zn, -ve), positive control (20 μM Zn, +ve), isotopically light (20 μM, ^64^Zn) and isotopically heavy (20 μM, ^68^Zn) exposures and grown under normal oxygen conditions. The cells were counted at 48, 72, and 96 h using a Cellometer Auto T4 (Nexcelom Bioscience, MA, US) to monitor the growth rate. To ensure constant cell proliferation the exposure media were refreshed after 48 h for the cells growing for 72 or 96 h.

### Uptake-Efflux Experiment

#### Cell Culturing

In our uptake-efflux experiment, MDA-MB-231 (ATCC HTB-26) cells were first cultured under sterile conditions inside a class II biological safety cabinet at 37°C. Cells were grown in Dulbecco's modified Eagle's medium (DMEM) + 10% fetal calf serum (FBS, Gibco Life, heat inactivated) + 1% penicillin/streptomycin (Sigma-Aldrich, UK) without Zn addition. After 48 h, the cells were transferred to petri dishes containing medium without additional Zn. At this step cells were grown on microscopic slides until they reached 10^5^ cells/cm^2^. Microscopic slides with MDA-MB-231 were transferred to media spiked with 15 μM isotopically natural Zn solution. A concentration of 15 μM ZnCl_2_ was selected as it is within the reference range of intracellular and plasma homeostatic Zn (11–24 μM) (41). As “free” Zn is toxic to cells, even at nanomolar levels (42), we added Zn complexed with histidine (Zn-His), a stable octahedral compound (43). MDA-MB-231 cells on microscopic slides were incubated for 24 h at 37°C before being transferred to the drip flow biofilm reactor for the efflux experiment.

#### Efflux Experiment

**Figure 2:**
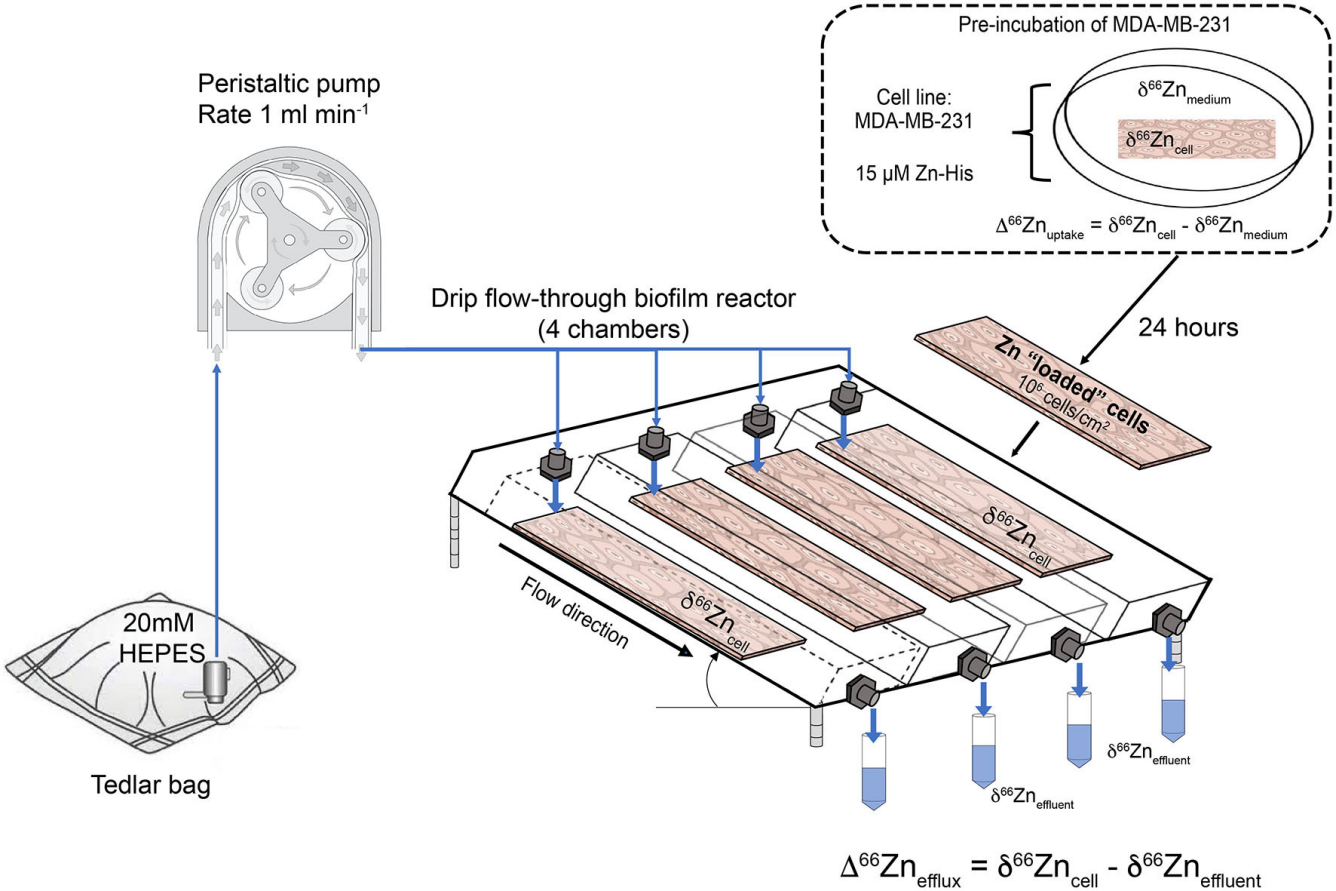
Schematic of the cell uptake-efflux experiment. A HEPES solution from the Tedlar bags is pumped to the drip flow-through reactor (1 ml min^−1^, as controlled by a peristaltic pump). The drip flow-through reactor contains 4 microscopic slides with MDA-MB-231 cells pre-grown in medium spiked with 15 μM Zn-histidine (Zn-His). Effluent samples were taken for 0.5 h in metal-free 50 mL centrifuge tubes.

Figure 2 shows the experimental setup for the efflux experiment using a drip flow-through biofilm reactor (BioSurface Technologies, Corp., USA). The experiment was conducted in a class II biological safety cabinet. Prior conducting the efflux experiment with a cell line, the setup was tested for Zn blanks using a 20 mM metal-free HEPES input solution. The polysulfone drip flow-through biofilm reactor has a 10° inclination and consists of four chambers. The 20 mM metal-free HEPES input fluid was supplied through four channels imposed by a peristaltic pump and ran through the system for 1 and 2.5 h, respectively, and the effluent was collected separately for each channel.

For the cell line efflux experiment, microscopic slides with cells cultured for 24 h in Zn-His spiked medium were washed three times with 20 mM metal-free HEPES solution before placed in the drip flow-through biofilm reactor to remove detached cells. This step ensured that the collected effluent solution of the efflux experiment only contained Zn excreted by the cells. The initial cell density and initial Zn isotope composition of these cells prior to the efflux experiments were determined by harvesting the cells from a microscopic slide with trypsin (Lonza). The harvested cells were counted using the Cellometer Auto T4 cell counter (Nexcelom, Bioscience). The 20 mM metal-free HEPES solution was directly dripped onto the cells with a rate of 1 ml min^−1^. Due to inclination of the drip flow-through reactor the residence time of the fluid passing the chambers was extremely short (<1 min) resulting in short contact time of the fluid and the cells. The effluent was collected in 30 min intervals in acid-washed metal-free centrifuge tubes (VWR). After the experiment, the cells on the microscopic slides were harvested using trypsin. Harvested cells, effluent and aliquots of the medium, and starting input HEPES solution were stored at −20°C, until required.

Prior to isotope analysis, harvested cells were lysed using a MARS 5 Digestion Microwave System (CEM Corp., UK). The harvested cell suspension was transferred in acid cleaned XP-1500 Plus (PTFE) vessels and 3 ml of quartz sub-boiled distilled nitric acid (15.4 N) and 2 ml hydrogen peroxide (Romil Ltd) were added. A blank sample consisting of only 3 ml of nitric acid and 2 ml hydrogen peroxide was processed for quality control. The samples were pre-digested at room temperature overnight. Microwave-assisted acid digestion was performed by ramping up the temperature stepwise to 210°C and 250 psi over 60 min, and held there for 30 min to ensure complete digestion.

For Zn isotope analysis, all samples (e.g., acid-digested cells, efflux solution) were double-spiked consisting of mixture of ^64^Zn and ^67^Zn. The double-spike mixture had a ^64^Zn/^67^Zn = 4.2336 and was added to each sample in an ideal sample/spike mixture of 1. To ensure the ideal sample/spike mixture, a small sample aliquot (20 μl) was spiked with an appropriate volume of ^64^Zn + ^67^Zn spike and the sample Zn concentration was determined by isotope dilution. Once sample-Zn was known, a larger aliquot of the sample was prepared for Zn isotopic analysis. Zinc was separated and purified using AG-MP1 resin (BioRad, 100–200 mesh) following the method described by (1, 44). The Zn columns with 250 μl AG-MP1 resin were rinsed with 10 mL of each 0.1N HNO_3_ and double-deionized water, conditioned with 6N HCl, and equilibrated with 4 x 0.5 ml 1N HCl. The spiked sample re-dissolved in 1 ml 1N HCl was loaded on the column and subsequently rinsed with 8 mL of 1N HCl. In the last step, 6 mL of 0.01N HCl was added to elute Zn from the column. Procedural blanks and Zn standard solutions (IRMM-3702, London-Zn) were processed in the same way as samples. The Zn isotope ratios and precise elemental Zn concentrations were measured with Nu Plasma HR-multiple-collector ICP-MS by double-spike and sample-standard bracketing techniques. All Zn isotope values were expressed as delta notation [δ^66^Zn (‰)] relative to the JMC-Lyon:


(1) δ66Zn(‰) =((Z66nZ64n)sample(Z66nZ64n)JMC−Lyon−1) × 1000

The extent of isotope fractionation was determined and described as:


(2) $$\begin{array}{l} \Delta^{66}\text{Z}{\text{n}}_{\text{uptake}}=\;{\text{δ}}^{66}\text{Z}{\text{n}}_{\text{cells}}-{\text{δ}}^{66}\text{Z}{\text{n}}_{\text{medium}} \end{array}$$

where Δ^66^Zn_uptake_ reflects Zn isotope fractionation for Zn taken up by cells (δ^66^Zn_cells_) from Zn-His spiked medium (δ^66^Zn_medium_) after incubation for 24 h, and Zn isotope fractionation of the efflux (Δ^66^Zn_efflux_) is described as:


(3) $$\begin{array}{l} \Delta^{66}\text{Z}{\text{n}}_{\text{efflux}}={\text{δ}}^{66}\text{Z}{\text{n}}_{\text{cells}}-{\text{δ}}^{66}\text{Z}{\text{n}}_{\text{effluent}} \end{array}$$

based on the difference between Zn isotopic composition of cells after incubation for 24 h (δ^66^Zn_cells_) and the Zn isotopic composition of the effluent (δ^66^Zn_effluent_) collected for 0.5 h.

The in-house London Zn was used as standard for sample-standard bracketing and the precision of the unprocessed London Zn solution was ± 0.04‰ (*n* = *26*) over two analytical days. The precision of δ^66^Zn for processed pure Zn IRMM-3702 reference material was −0.13 ± 0.08‰ (2SD, *n* = *6*). If not stated otherwise, the precision of 2SD for δ^66^Zn (‰) refers to repeated analysis of London Zn bracketing standards.

## Results

### Zinc Isotope Enrichment Proliferation Tests

**Figure 3:**
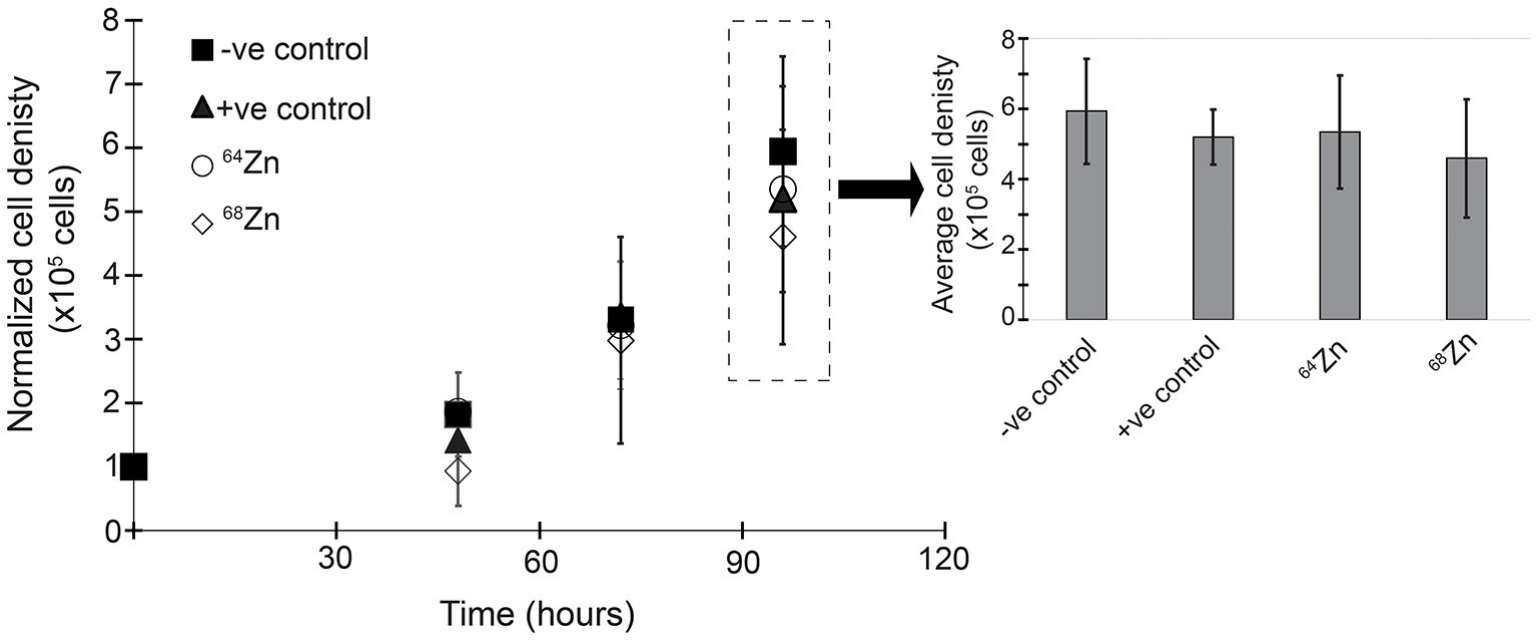
Zinc isotope enrichment proliferation after 96 h under different medium Zn conditions: -ve control = negative control without Zn; +ve control = positive control with 20 μM “natural” Zn; 64-Zinc = 20 μM ^64^Zn and 68-Zinc = 20μM ^68^Zn only.

Figure 3 shows the average cell growth of MDA-MB-231 in a ^64^Zn and ^68^Zn isotope enriched medium as well as the control without Zn (-ve) and natural Zn (+ve). In all experiments, regardless of the isotopic composition of the Zn in solution, the cell density increased by 5-fold within 96 h reaching an average density of 5.3 × 10^5^ ± 0.5 × 10^5^ cells. If cell growth is driven by the mass of an isotope, we would expect to see faster growth for the lighter isotopes compared t the heavier isotopes of Zn (Figure 3 and Table 1).

**Table 1:** Data summary for Zn isotope enrichment proliferation tests.

**Experimental** **condition**	**Time** **(hrs)**	**Average** **(x10^**5**^ cells)**	**Stdev** **(x10^**5**^ cells)**
-ve control	0	1.00	–
-ve control	48	1.82	0.66
-ve control	72	3.30	0.93
-ve control	96	5.94	1.49
+ve control	0	1.00	–
+ve control	48	1.42	0.40
+ve control	72	3.39	1.74
+ve control	96	5.20	0.79
^64^Zn	0	1.00	–
^64^Zn	48	1.86	0.05
^64^Zn	72	3.21	0.99
^64^Zn	96	5.35	1.61
^68^Zn	0	1.00	–
^68^Zn	48	0.93	0.54
^68^Zn	72	2.98	1.62
^68^Zn	96	4.60	1.68
-ve control	96	5.94	1.49
+ve control	96	5.20	0.79
^64^Zn	96	5.35	1.61
^68^Zn	96	4.60	1.68

### Cell Efflux

*Control of blank levels*. We assessed blank contributions because Zn usually has a high background level, which can significantly alter the measured isotopic composition. The microwave control blank was 0.9 ng Zn ml^−1^ and procedural blanks for the anion exchange chromatography were 0.24 ± 0.20 ng Zn ml^−1^ (*n* = *4*). The Zn blank for the experimental setup ranged between 0.5 and 2.7 ng Zn ml^−1^ after 1 h effluent collection and decreased to 0.6–1.4 ng Zn ml^−1^ after 2.5 h (Table 2). This corresponds to 0.7–4.8% of Zn (mean: 1.9 ± 2.0% Zn) collected in the effluent of the cell line efflux experiment. The sum of all blanks (microwave, procedural and efflux) corresponds to 0.008 and 0.81% of the initial Zn in medium and harvested cells before the efflux experiment. This observation demonstrates that precise and reliable Zn isotope data can be achieved for cell line experiments using the drip flow-through biofilm reactor.

**Table 2:** Data summary for uptake-efflux experiment.

**Experiment**	**Sample** **type**	**Time** **(h)**	**Flow reactor** **channel**	**Zn concentration** **(ng/ml)**	**% Zn rel.** **efflux**	**Number of** **analysis**
Blank test	Fluid	1	1	NA		–
	Fluid	1	2	0.48		1
	Fluid	1	3	2.09		1
	Fluid	1	4	2.66		1
Blank test	Fluid	2.5	1	0.68		1
	Fluid	2.5	2	1.38		1
	Fluid	2.5	3	0.55		1
	Fluid	2.5	4	NA		–
**Experiment**	**Sample type**	**Time (h)**	**Flow reactor channel**	**Zn concentratio*****n*** **(ng/ml)**	**δ^66^Zn**_**IRMM3702**_ **(**‰**)**	**Number of analysis**
Efflux	Fluid	0.5	1	105	−0.19 (0.05)	1
	Fluid	0.5	2	40	−0.07 (0.04)	1
	Fluid	0.5	3	70	−0.11 (0.05)	1
	Fluid	0.5	4	55	NA	1
Initial Zn medium	Fluid	0			−0.03 (0.07)	2
Cells before efflux	Solid	–		302	+0.2 (0.07)	1
IRMM-3702 (processed)					−0.13 (0.08)	6

**Figure 4:**
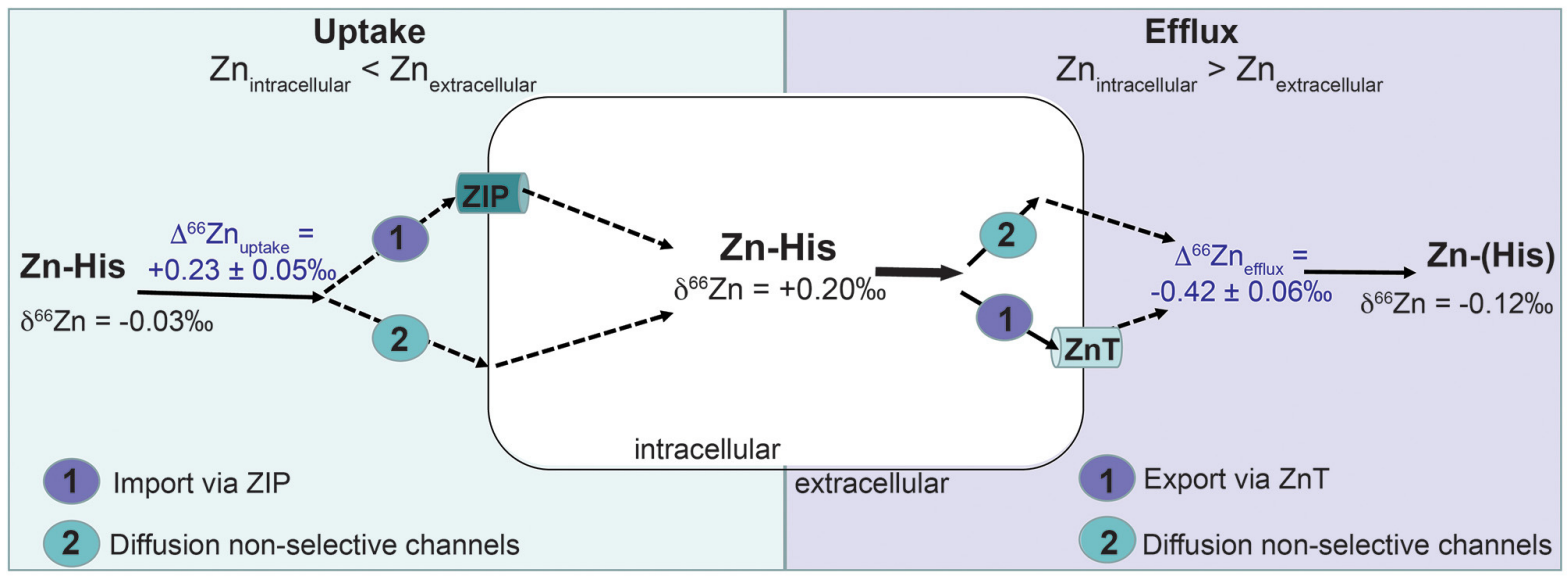
Possible pathways that may lead to Zn isotope fractionation (Δ^66^Zn) based on the uptake-efflux experiment with the MDA-MB-231 cell line. Zn was provided as Zn complexed with histidine (Zn-His). Zn isotope fractionation by cell uptake (Δ^66^Zn_uptake_) can be caused by (1) active import of Zn-His via Zn importer proteins (ZIP) or (2) non-quantitative diffusion of Zn via unspecified channels. Zn isotope fractionation via efflux (Δ^66^Zn_efflux_) of Zn out of the cells can be caused by (1) active export of Zn-His via Zn transporter proteins (ZnT) or (2) non-quantitative diffusion of Zn via unspecified channels.

*Mass balance*. Our current experimental design does not allow for a complete isotopic mass balance. Cellular concentrations and isotopic changes of Zn can only be monitored assuming a similar distribution and behavior of cells on each microscopic slide. Based on the Zn concentration of harvested cells from two microscopic slides, we calculated that about 0.15% of total 15 μM Zn from the medium was incorporated into MDA-MB-231 cells. With an initial MDA-MB-231 cell density of 2.6 × 10^6^ ± 0.62 × 10^6^ cells (*n* = *2*), the Zn uptake yielded in 8.9 × 10^−6^ nM Zn/cell (= accounting for 5.4 × 10^9^ Zn atoms/cell). The initial medium spiked with isotopic naturally-distributed 15 μM Zn-His had a δ^66^Zn of −0.03 ± 0.02‰ (2 SD, *n* = *2*). MDA-MB-231 cells treated with 15 μM Zn-His had a △^66^Zn of +0.20‰ prior to the efflux experiment. The intracellular Zn of MDA-MB-231 is isotopically heavier compared to the initial medium (Δ^66^Zn_uptake_ = +0.23 ± 0.1‰; 2SD, Figure 4). Zinc in the effluent collected from four channels corresponds to 4.03 ± 1.66 × 10^−7^ nM Zn/cell (= accounting for 2.4 × 10^8^ Zn atoms/cell). The δ^66^Zn of the efflux solutions collected from the four channels range between −0.07 and −0.19‰ with an average of −0.12 ± 0.12‰ (2 SD).

## Discussion

The results show nearly identical proliferation of MDA-MB-231 under single isotope and natural Zn exposure conditions; these observations demonstrate that isotopic mass does not control cell growth. The addition of 20 μM Zn did not enhance or suppress the proliferation, as the cell density for light (^64^Zn) or heavy (^68^Zn) isotopically spiked Zn conditions are similar to those for the no-Zn control and Zn with natural isotope composition (Figure 3).

Metabolism in cancerous breast tissue is generally thought to be enhanced in order to provide sufficient energy and anabolic substrate for proliferation (45, 46). Adaptation in Zn-protein expression in neoplasms is not driven by preferential uptake of any Zn isotopologue. This means that the isotopic shift reported in breast tumors (3, 5) likely reflects metabolic changes for Zn in cancerous breast cells, and not an energetic advantage related to the isotopic composition possessed by the neoplasm. As such, observed isotopic shifts in breast cancer tissue can be used purely as a monitor of neoplastic driven metabolic changes. Furthermore, single stable isotope tracer studies are established as providing better insight into the processes under investigation without inducing or exacerbating pathological processes (47).

Notably, our MDA-MB-231 cell line analyses show isotopic fractionations in the opposite direction to those determined for *in vivo* breast cancer tissue samples. Malignant breast tumors have significantly lower δ^66^Zn than healthy tissues which has been interpreted as an indicator for increased metallothionein expression (3, 5). The difference in Zn isotope fractionation observed between *in vitro* MDA-MB-231 cell line experiments and *in vivo* breast tissue might be due to differences in Zn transporter levels or intercellular Zn storage mechanisms (e.g., metallothionein expression).

A positive direction of Zn isotope fractionation for the cell uptake (Δ^66^Zn_uptake_ = +0.23 ± 0.1‰, 2SD, *n* = *2*) can be mechanistically explained by Zn adsorption, diffusion through non-selective channels into the cell or an active cell uptake via ZIP proteins. Adsorption studies on bacterial cells and biofilms show preferential binding of heavy Zn isotopes with equilibrium isotopic fractionation between +0.50 and +1.30‰ (48, 49). However, adsorption may be less likely in our studies as the 2:1 Zn (His)_2_ complexes are uncharged (43) and cells were rinsed with metal-free HEPES to remove any loosely adsorbed Zn. Instead, the preferential uptake of ^66^Zn by MDA-MB-231 may reflect previously reported upregulation of histidine-rich ZIPs in breast cancer cells (25–29). ZIP proteins (ZIP1-ZIP14) control intracellular Zn levels by transporting Zn into the cells across the cell membrane. Upregulation of ZIPs has been observed in most cancers to meet the demand of increased rate of proliferation and metabolism (46). An indicator for ZIP upregulation is one-order of magnitude higher Zn uptake of 5.4 × 10^9^ Zn atoms/cells by MDA-MB-231 in our experiments compared to Zn required by mammalian cells (10^8^ Zn atoms/cell) (50).

Zinc isotope fractionation for Zn uptake by cells can be associated with Zn speciation, Zn-binding domains of proteins or exchange reactions. A study with neuron-like cells showed a preferential uptake of light ^64^Zn with an isotopic fractionation of 1.14‰ (39). However, uptake and efflux of Zn-His should not fractionate Zn isotopes due to a lack in coordination change for complexed Zn species (51). Caldelas and Weiss (51), however, argued that non-quantitative uptake of complexed Zn can result in an enrichment of ^66^Zn in plant cells. We can assume a similar isotopic effects of non-quantitative uptake for Zn-His because only 0.15% of Zn from the medium has been taken up by MDA-MB-231 cells. However, our data cannot directly resolve whether diffusion through non-selective channels or uptake via ZIPs, or other proteins, is the predominant pathway for Zn uptake by MDA-MB-231 cells.

Negative Δ^66^Zn_efflux_ suggests diffusion via non-selective channels. During diffusion light ^64^Zn diffuses faster out of the cell than ^66^Zn. The efflux experiment shows that MDA-MB-231 cells preferentially 'excrete' light ^64^Zn with Δ^66^Zn_efflux_ of −0.42 ± 0.12‰ (Figure 4). An existing concentration gradient with high Zn in cells and no Zn in the efflux solution may allow diffusion of Zn-His via non-selective channels. Coutaud et al. (49) reported preferential excretion of lighter Zn isotopes with Δ^66^Zn of −0.50 ± 0.20‰ due to desorption from the biofilm. Also, lighter Zn isotopes can diffuse faster through biological membranes or due to a gradient within the boundary layer (52). This leaves the cell culture enriched in heavy ^66^Zn over time resulting in opposite isotopic signal of what has been observed for *in vivo* malignant breast tissue. These *in vivo* studies found that Zn isotope compositions are about 0.17‰ lower in malignant breast than healthy tissues (3, 5).

The ZnTs family (ZnT1-ZnT10) regulates intracellular Zn levels by transporting Zn from the cytoplasm into the extracellular space. To decrease the toxic effects of high Zn in breast cancer cells, ZnT1 is overexpressed to prevent apoptotic cell death and increase Zn secretion. Wang et al. (53) observed that the mRNA expression level of ZnT-1 increases significantly in MDA-MB-231 cells when they are exposed to Zn sulfate. *In vivo*, ZnTs transport only free Zn^2+^ across cell membranes (54) and with that most likely isotopically heavy Zn^2+^ out of the cell due to their histidine-rich structure. In our experiments, however, Zn-His might be able to pass cell membranes via different channels and the diffusion out of the cell is similar to Zn-His uptake. This explains the preferential efflux of light ^64^Zn due to faster diffusion of light ^64^Zn. Obviously conducting an uptake-efflux experiment with free Zn^2+^ could be a better approach, but even if Zn is added to the medium as free Zn^2+^, culture medium contains millimolar levels of ligands (e.g., histidine, cysteine and phosphate) which rapidly complex free Zn^2+^ (42). Moreover, conducting an experiment with “free” Zn can result in apoptosis because “free” Zn is a cell toxin at even nanomolar levels (42).

*In vivo*, tumors are heterogenous, have a three-dimensional shape and are often mixed with connective tissue, immune cells, and stromal tissues (55–57). More specifically, breast cancer varies in its origin and genetic lesions, leading to distinct phenotypes (58, 59) which is also reflected in different ZIP and ZnT expression between various breast cancer cell lines. Therefore, we tested our findings from MDA-MB-231 in a large panel of breast cancer cell lines (60) to further our understanding in cell lines representing patient subtypes (59) (Table 3). Using a well-defined list of human ZnT and ZIP proteins (23), we identified ten Zn proteins which are differentially expressed (FDR-adjusted *P* < 0.1) in MDA-MB-231 compared to other basal-like breast cancer cell lines (e.g., Her2, Luminal A and Luminal B) (Table 3). It shows that more ZIPs and ZnTs are up- than downregulated which might explain the high uptake and efflux. Other studies on the hormone-dependent breast cancer cell lines T-47D and MCF-7 showed that ZnT2 and metallothionein are overexpressed, providing protection from Zn hyperaccumulation and preventing apoptosis by either removing Zn from the cell or redistributing it among cellular compartments (27, 29). Consequently, whether breast cancer cell lines and the experimental approach with initially ligand complexed Zn (Zn-His) reflect that of *in vivo* carcinoma remains an important issue to resolve before drawing any reliable conclusion on Zn isotope fractionation in clinical samples.

**Table 3:** Differential gene expression analysis for ZnTs and ZIPs between the basal-like (including MDA-MB-231) and other breast cancer cell lines (Her2, Luminal A and Luminal B).

**Protein coding** **gene**	**Proteins**	**logFC**	***P*-value**	**Q-value**	**Direction** **of expression**
SLC39A6	ZIP6	−1.172	0.001	0.007	Down
SLC39A9	ZIP9	−0.521	0.000	0.001	Down
SLC39A11	ZIP11	−0.899	0.000	0.003	Down
SLC30A9	ZnT9	−0.318	0.036	0.095	Down
SLC39A4	ZIP4	0.899	0.012	0.041	Up
SLC39A8	ZIP8	0.952	0.010	0.040	Up
SLC39A10	ZIP10	0.430	0.040	0.095	Up
SLC39A14	ZIP14	0.938	0.010	0.040	Up
SLC30A3	ZnT3	0.769	0.006	0.033	Up
SLC30A6	ZnT6	0.243	0.036	0.095	Up
SLC30A5	ZnT5	−0.226	0.068	0.148	Normal
SLC30A10	ZnT10	0.113	0.079	0.158	Normal
SLC39A12	ZIP12	0.021	0.137	0.254	Normal
SLC39A2	ZIP2	0.373	0.153	0.262	Normal
SLC30A4	ZnT4	−0.207	0.320	0.510	Normal
SLC39A7	ZIP7	−0.164	0.356	0.510	Normal
SLC39A1	ZIP1	−0.162	0.361	0.510	Normal
SLC39A13	ZIP13	−0.194	0.419	0.558	Normal
SLC30A8	ZnT8	0.146	0.522	0.659	Normal
SLC30A2	ZnT2	0.095	0.555	0.667	Normal
SLC39A5	ZIP5	−0.027	0.653	0.706	Normal
SLC30A7	ZnT7	0.062	0.687	0.706	Normal
SLC39A3	ZIP3	0.061	0.701	0.706	Normal
SLC30A1	ZnT1	0.081	0.706	0.706	Normal

*FC, fold change; For each gene, log2FC was calculated as the difference in means of mRNA abundance between the basal-like and other cell lines. Welch's t-test was used to quantify statistical significance in the difference in mRNA abundance of the two groups. P-values were adjusted for multiple comparisons (Q-values) using the Benjamini-Hochberg method. P-values < 0.05 are considered being significant. Blue: Downregulated ZnTs and ZIPs in MDA-MB-23 relative to other basal-like breast cancer cell lines. Green: Upregulated ZnTs and ZIPs in MDA-MB-23 relative to other basal-like breast cancer cell lines*.

### Future Directions

Our results support the relevance of *in vitro* cell culture models to identify and understand factors driving changes in Zn isotopic composition caused by cancer development and progression. However, with respect to *in vivo* relevance, the results should be regarded as preliminary, and additional experiments are required using other human breast cancer cell lines, e.g., MCF-7, BT-20, with different expressions of ZnT and ZIP (Table 1). This is important, because, for instance, upregulation of ZIP6 in estrogen treated MCF-7 (61) (Table 1) might lead to more pronounced uptake of heavy ^66^Zn. As almost every cancer is unique, it may be advantageous to determine average Zn isotope fractionation for cancer cells based on Zn biology (e.g., MT expression, ZnT and ZIP regulation, cell growth rate) moreover the use of 3D cell cultures as spheroids may be a representative approach and enable other stromal cell types to be incorporated into the workstream.

## Data Availability Statement

The raw data supporting the conclusions of this article will be made available by the authors, without undue reservation.

## Author Contributions

KS and FL designed the experiments. KS performed the data analysis, interpretation of the data, and drafting of the manuscript. ALH provided lab space at MRC Weatherall Institute of Molecular Medicine to conduct the uptake-efflux experiment. The isotopic analyses were conducted in the Oxford Earth Sciences isotope facility of ANH. SH provided the bioinformatic data and wrote the bioinformatic parts of the manuscript. All authors contributed to the article and approved the submitted version.

## Funding

This work was funded by Cancer Research UK (No. C5255/A18085), through the CRUK Oxford Centre and John Fell Oxford University Press Research Fund (No. 153/035).

## Conflict of Interest

The authors declare that the research was conducted in the absence of any commercial or financial relationships that could be construed as a potential conflict of interest.

## Publisher's Note

All claims expressed in this article are solely those of the authors and do not necessarily represent those of their affiliated organizations, or those of the publisher, the editors and the reviewers. Any product that may be evaluated in this article, or claim that may be made by its manufacturer, is not guaranteed or endorsed by the publisher.

## References

[1] Schilling K Larner F Saad A Roberts R Kocher HM Blyuss O. Urine metallomics signature as an indicator of pancreatic cancer. Metallomics. (2020) 12:752–7. 10.1039/d0mt00061b 32211672

[2] Schilling K Moore RET Sullivan KV Capper MS Rekämper M Goddard K. Zinc stable isotopes in urine as diagnostic for cancer of secretory organs. Metallomics. (2021) 13:mfab020. 10.1093/mtomcs/mfab020 33877364

[3] Sullivan K Moore RET Capper MS Schilling K Goddard K Ion C. Zinc stable isotope analysis reveals Zn dyshomeostasis in benign tumours, breast cancer, and adjacent histologically normal tissue. Metallomics. (2021) 13:mfab027. 10.1093/mtomcs/mfab027 33970272

[4] Balter V Nogueira de Costa A Bondanese VP Jaouen K Lamboux A Sangrajrang S. Natural variations of copper and sulfur stable isotopes in blood of heptacellular carcinoma patients. Proc Natl Acad Sci. (2015) 112:982–5. 10.1073/pnas.1415151112 25583489 PMC4313854

[5] Larner F Woodley LN Shousha S Moyes A Humphreys-Williams E Strekopytov S. Zinc isotopic compositions of breast cancer tissue. Metallomics. (2015) 7:112–7. 10.1039/C4MT00260A 25489714

[6] Telouk P Puisieux A Fujii T Blater V Bondanese VP Morel. Copper isotope effect in serum of cancer patients. A pilot study. Metallomics. (2015) 7:299–308. 10.1039/C4MT00269E 25532497

[7] Anoshika Y Costas-Rodriguez M Speeckaert M Van Biesen W Delanghe J Vanhaecke F. Iron isotopic composition of blood serum in anemia of chronic kidney disease. Metallomics. (2017) 9:517–24. 10.1039/C7MT00021A 28417130

[8] Sauzéat L Bernard E Perret-Liaudet A Quardrio I Vighetto A Krolak-Salmon P. Isotopic evidence for disrupted copper metabolism in amyotrophic lateral sclerosis. Science. (2018) 6:264–71. 10.1016/j.isci.2018.07.023 30240616 PMC6137708

[9] Moore RET Rehkämper M Maret W Larner F. Assessment of coupled Zn concentration and natural stable isotope analyses of urine as a novel probe of Zn status. Metallomics. (2019) 11:1506–17. 10.1039/c9mt00160c 31411226

[10] Hastuti AAMB Costas-Rodriguez M Matsunaga A Ichinose T Hagiwara S. Cu and Zn isotope variations in plasma for survival prediction in hematological malignancy cases. Sci Rep. (2020) 10:16389. 10.1038/s41598-020-71764-7 33009454 PMC7532200

[11] Moynier F Le Borgne M Lahoud E Mahan B Mouton-Ligier F Hugon J. Copper and zinc isotopic excursions in the human brain affected by Alzheimer's disease. DADM. (2020) 12:e12112. 10.1002/dad2.12112 33102682 PMC7571480

[12] Toubhans B Gourlan AT Telouk P Lutchman-Singh K Francis LW Conlan RS. Cu isotope ratios are meaningful in ovarian cancer diagnosis. J Trace Elem Med Biol. (2020) 62:126611. 10.1016/j.jtemb.2020.126611 32652467

[13] Van Campenhout S Hastuti A.A.M.B. Lefere S Van Vlierberghe H Vanhaecke F. Lighter serum copper isotopic composition in patients with early non-alcoholic fatty liver disease. BMC Res Notes. (2020) 13:225. 10.1186/s13104-020-05069-3 32306999 PMC7168815

[14] Solovyev N El-Khatib A.H. Costas-Rodriguez M Schwab K Griffin E. Cu, Fe, Zn isotope ratios in murine Alzheimer's disease models suggest specific signatures of amyloidogenesis tauopathy. J Biol Chem. (2021) 296:100292. 10.1016/j.jbc.2021.100292 33453282 PMC7949056

[15] Maret W. Human zinc biochemistry. In: Rink L, editors. Zinc in Human Health. Aachen: IOS Press (2011). p. 45–62.

[16] Kambe T Hashmoto A Fujimoto S. Current understanding of ZIP and ZnT zinc transporters in human health and diseases. Cell Mol Life Sci. (2014) 71:3281–95. 10.1007/s00018-014-1617-0 24710731 PMC11113243

[17] Hara T Takeda T Takagishi T Fukue K Kambe T Fukada T. Physiological roles of zinc transporters: molecular genetic importance in zinc homeostasis. J Physiol Sci. (2017) 67:283–301. 10.1007/s12576-017-0521-4 28130681 PMC10717645

[18] Maret W. Zinc in cellular regulation: the nature and significance of Zinc signals. Int J Mol Sci. (2017) 18:2285. 10.3390/ijms18112285 29088067 PMC5713255

[19] Maret W. Regulation of cellualr zinc ions and their signaling functions. Cell Signal. (2019) 2019:5–22. 10.1007/978-981-15-0557-7_2

[20] Potocki S Valensin D Kozlowski H. The specificity of interaction of Zn^2+^, Ni^2+^ and Cu2+ ions with the histidine-rich domain of the TjZNT1 ZIP family transporter. Dalton Trans. (2014) 43:10215–23. 10.1039/C4DT00903G 24874820

[21] Maret W. The function of zinc metallothionein: a link between cellular zinc redox state. J Nutr. (2000) 130:1455S–8S. 10.1093/jn/130.5.1455S 10801959

[22] Krezel A Maret W. The function of metamorphic metallothioneins in zinc copper metabolism. Int J Mol Sci. (2017) 18:1237. 10.3390/ijms18061237 28598392 PMC5486060

[23] Bafaro E Liu Y Xu Y Dempski RE. The emerging role of zinc transporters in cellular homeostasis and cancer. Sign Transduct Target Therapy. (2017) 2:17029. 10.1038/sigtrans.2017.29 29218234 PMC5661630

[24] Maret W. Zinc coordination environments in proteins determine zinc functions. J Trace Elem Med Biol. (2005) 19:7–12. 10.1016/j.jtemb.2005.02.003 16240665

[25] Kagara N Tanaka N Noguchi S Hirano T. Zinc and its transporter ZIP10 are involved in invasive behavior of breast cancer cells. Canc Sc. (2007) 98:692–7. 10.1111/j.1349-7006.2007.00446.x 17359283 PMC11159674

[26] Taylor KM Vichova P Jordan N Hiscox S Hendley R Nicholson RI. ZIP7-mediated intracellular zinc transport contributes to aberrant growth factor signaling in antihormone-resistant breast cancer cells. Endocrinolog. (2008) 149:4912–20. 10.1210/en.2008-0351 18583420

[27] Lopez V Foolad F Kelleher SL. ZnT2-overexpression represses the cytotoxic effects of zinc hyper-accumulation in malignant metallothionein-null T47D breast tumor cells. Canc Lett. (2011) 304:41–51. 10.1016/j.canlet.2011.01.027 21353385

[28] Takatani-Nakase T. Zinc transporters and progression of breast cancer. Biol Pharm Bull. (2018) 41:1517–22. 10.1248/bpb.b18-00086 30270320

[29] Alam S Kelleher SL. Cellular mechanisms of zinc dysregulation: a perspective on zinc homeostasis as an etiological factor in the development and progression of breast cancer. Nutrients. (2012) 4:875–903. 10.3390/nu4080875 23016122 PMC3448077

[30] Moynier F Fuji T Shaw AS Le Borgne M. Heterogenous distribution of natural zinc isotopes in mice. Metallomics. (2013) 5:693–9. 10.1039/c3mt00008g 23589059

[31] Markovic T Manzoor S Humphreys-Williams E Kirk GJD Vilar R Weiss DJ. Experimental determination of zinc isotope fractionation in complexes with the phytosiderophore 2'-deoxymugeneic acid (DMA). and its structural analogues, and implications for plant uptake mechanisms. Environ Sci Technol. (2017) 51:98–107. 10.1021/acs.est.6b00566 27750003

[32] Fujii T Albarède F. Ab Initio Calculation of the Zn Isotope Effect in Phosphates, Citrates, and Malates and Applications to Plants and Soil. PLoS ONE (2012) 7:e30726. 10.1371/journal.pone.0030726 22363478 PMC3281869

[33] Fujii T Moynier F Blichert-Toft J Albarède F. Density functional theory estimation of isotope fractionation of Fe, Ni, Cu, and Zn among species relevant to geochemical and biological environments, Geochim. Cosmochim Acta. (2014) 140:553–76. 10.1016/j.gca.2014.05.051

[34] Stenberg A Malinovsky D Öhlander B Andrén H Forsling W Engström LM. Measurement of iron and zinc isotopes in human whole blood: Preliminary application to the study of HFE genotypes. J Trac Elem Med Biol. (2005) 19:55–60. 10.1016/j.jtemb.2005.07.004 16240673

[35] Flórez MR Costas-Rodriguez M Grootaert C Van Camp J Vanhaecke F. Cu isotope fractionation response to oxidative stress in hepatic cell line studied using multi-collector ICP-mass spectrometry. Anal Bioanal Chem. (2018) 410:2385–94. 10.1007/s00216-018-0909-x 29404664

[36] Costas-Rodriguez M Colina-Vegas L Solovyev N De Wever O Vanhaecke F. Cellular and sub-cellular Cu isotope fractionation in the human neuroblastoma SH-SY5Y cell line: proliferating versus neuron-like cells. Anal Bioanal Chem. (2019) 411:4963–71. 10.1007/s00216-019-01871-6 31093701

[37] Flórez MR Anoshkina Y Costas-Rodriguez M Grootaert C Van Camp J Delanghe J. Natural Fe isotope fractionation in an intestinal Caco-2 cell line model. J Anal At Spectrom. (2017) 32:1713–20. 10.1039/C7JA00090A

[38] Paredes E Avazeri E Malard V Vidaud C Reiller PE Ortega R. Impact of uranium uptake on isotopic fractionation and endogenous element homeostasis in human neuron-like cells. Sci Rep. (2018) 9:17163. 10.1038/s41598-018-35413-4 30464301 PMC6249223

[39] Paredes E Avazeri E Malard V Vidaud C Reiller PE Ortega R. Evidence of isotopic fractionation of natural uranium in cultured human cells. Proc Natl Acad Sci USA. (2016) 49:14007–12. 10.1073/pnas.1610885113 27872304 PMC5150385

[40] Bondanese VP Lamboux A Simon M Lafontm JE Albalat E Pichat S. Hypoxia induces copper stable isotope fractionation in hepatocellular carcinoma, in a HIF-independent manner. Metallomics. (2016) 8:1177–84. 10.1039/C6MT00102E 27500357

[41] Taylor A. Detection and monitoring of disorders of essential trace elements. Ann Clin Biochem. (1996) 33:486–510. 10.1177/000456329603300603 8937580

[42] Bozym RA Chimienti F Giblin LJ Fross GW Korichneva I Li Y. Free zinc ions outside a narrow concentration range are toxic to a variety of cells in vitro. Exp Biol Med. (2010) 235:741–50. 10.1258/ebm.2010.009258 20511678 PMC2896872

[43] Krezel A Maret W. The biological inorganic chemistry of zinc ions. Anal Biochem Biophys. (2016) 611:3–19. 10.1016/j.abb.2016.04.010 27117234 PMC5120989

[44] Arnold T Schönbächler M Rehkämper M Dong S Zhao F.-J.. Measurement of zinc stable isotope ratios in biogeochemical matrices by double spike MC-ICPMS and determination of the isotope ratio pool available for plants from soil. Anal Bioanal Chem. (2010) 398:3115–25. 10.1007/s00216-010-4231-5 20890747 PMC2990013

[45] Gatenby RA Gillies RJ. Hypoxia and metabolism – opinion – a microenvironmental model carcinogenesis. Nat Rev Cancer. (2008) 8:56–61. 10.1038/nrc2255 18059462

[46] Pan Z Choi S Ouadid-Ahidouch H Yang JM Beattie JH Korichneva I. Zinc transporters and dysregulated channels in cancers. Front Biosci. (2017) 22:623–43. 10.2741/4507 27814637 PMC5199720

[47] Stürup S. The use of ICPMS for stable isotope tracer studies in humans: a review. Anal Bioanal Chem. (2004) 378:273–82. 10.1007/s00216-003-2195-4 13680059

[48] Kafantaris F-C Borrok DM. Zinc isotope fractionation during surface adsorption and intracellular incorporation by bacteria. Chem Geol. (2014) 366:42–51. 10.1016/j.chemgeo.2013.12.007 PMC3140435 21785492

[49] Coutaud A Meheut M Viers J Rols J-L Pokrovsky OS. Zn isotope fractionation during interaction with phototropic biofilm. Chem Geol. (2014) 390:46–60. 10.1016/j.chemgeo.2014.10.004

[50] Palmiter RD Findley SD. Cloning and functional characterization of a mammalian zinc transporter that confers resistance to zinc. EMBO J. (1995) 14:639–49. 10.1002/j.1460-2075.1995.tb07042.x 7882967 PMC398127

[51] Caledas C Weiss DJ. Zinc homeostasis and isotopic fractionation in plants: a review. Plant Soil. (2017) 411:17–46. 10.1007/s11104-016-3146-0

[52] Rodushkin I Stenberg A Andren H Malinovsky D Baxter DC. Isotopic fractionation during diffusion of transition metal ions in solution. Anal Chem. (2004) 76:2148–51. 10.1021/ac035296g 15053683

[53] Wang Y Li KJ. Mao L Zhao WJ.. Effects of exogenous zinc on cell cycle, apoptosis and viability of MDAMB231, HepG2 and 293 T cells. Biol Trace Elem Res. (2013) 154:418–26. 10.1007/s12011-013-9737-1 23839533

[54] Eide DJ. Zinc transporters and cellular trafficking of zinc. Biochim Biophys Acta. (2006) 1763:711–22. 10.1016/j.bbamcr.2006.03.005 16675045

[55] Beca F Polyak K. Intratumor heterogeneity in breast cancer. Adv Exp Med Biol. (2016) 882:169–89. 10.1007/978-3-319-22909-6_7 26987535

[56] Riesop D Hirner AV Rusch P Bankfalvi A. Zinc distribution within breast cancer tissue: a possible marker for histological grading? J Cancer Res Clin Oncol. (2015) 141:1321–31. 10.1007/s00432-015-1932-3 25672953 PMC11823795

[57] Rusch P Hirner AV Schmitz O Kimmig R Hoffmann O Diel M. Zinc distribution within breast cancer tissue of different intrinsic subtypes. Arch Gynecol Obst. (2020) 2020:1–11. 10.1007/s00404-020-05789-8 32930875 PMC7854450

[58] Turashvili G Brogi E. Tumor heterogeneity in breast cancer. Front Med. (2017) 4:227. 10.3389/fmed.2017.00227 29276709 PMC5727049

[59] Sørlie T Perou CM Tibshirani R Ass T Geisler S Johnsen H. Gene expression patterns of breast carcinomas distinguish tumor subclasses with clinical implications. Proc Natl Acad Sci USA. (2001) 98:10869–74. 10.1073/pnas.191367098 11553815 PMC58566

[60] Barretina J Caponigro G Stransky N Venkatesan K Margolin AA Kim S. The cancer cell line encyclopedia enables predictive modeling of anticancer drug sensitivity. Nature. (2012) 483:603–7. 10.1038/nature11003 22460905 PMC3320027

[61] Takatani-Nakase T Matsui C Maeda S Kawahara S Takahashi K. High glucose level promotes migration behavior of breast cancer cells through zinc and its transporters. PLoS ONE. (2014) 9:e90136. 10.1371/journal.pone.0090136 24587242 PMC3938647

